# Disease of the Heart

**Published:** 1855-03

**Authors:** 


ART. III.—*Disease of the Heart.* By R. S. L., Dubuque.

L. W. C., aged 23, a Dep. U. S. Surveyor, on the 29th of Nov. last made application to me for medical advice. I found on examination, a jaundiced condition of the system, with enlarged glands. The abdomen considerably distended from enlarged liver and spleen. The glands of the neck, axilla, and groin, very much enlarged; slightly tender to the touch. No feverish action, good appetite, bowels costive. Ordered Blue Mass and Tincture Cherry Bark.

Saw him again, Dec. 8th. Yellow appearance of skin disappearing. Ordered hyd. potas. and syr sarsap.; ung. iod. to enlarged glands. Saw him almost daily to 25th December, during which time the enlarged glands were rapidly disapppearing.

On the 27th was called to see him; found great difficulty in breathing; could not sleep except in a sitting posture; pulse 120, some cough, expectorating a bloody mucous, dullness over the apex of left lung, tumultuous action of the heart, and heard over a large space. Learned at this visit the patient, some few months previous, had an attack of Rheumatism; which, in connexion with present symptoms, seemed to point out a case of Hydrops Pericardium.

From this time, up to January 6th, when the patient died, he was actively treated with tinct. digitalis, tar. ant., win. of colch. blisters, &c., without diminishing the heart's action in the least. No feverish action was perceptible at any of the visits; and he complained of no pain, only a slight soreness through the chest. The expectoration changed but slightly during the short time, and was tolerably free.

Post mortem: Lungs healthy, except slight irritation of bronchial tubes, crowded into a small space. 16 oz. water in the pericardium. Heart hypertrophoid, weighing 2 lbs. after all the fluids were removed from it. Ventricle, and all the large vessels, filled with coagulable lymph. The left ventricle, half way between the base and apex, measured 1⅓ inches through its wall. Liver and spleen enlarged, but normal in appearance, the former weighing 6½ lbs the latter 3½. No appearance of recent inflammation, except in right auricle of the heart.

